# Predictive factors of *Clostridioides difficile* infection in hospitalized patients with new diarrhea: A retrospective cohort study

**DOI:** 10.1371/journal.pone.0207128

**Published:** 2018-12-05

**Authors:** Koray K. Demir, Matthew P. Cheng, Todd C. Lee

**Affiliations:** 1 Department of Medicine, McGill University Health Centre, Montréal, Canada; 2 Division of Infectious Diseases, Department of Medicine, McGill University Health Centre, Montréal, Canada; 3 Clinical Practice Assessment Unit, Department of Medicine, McGill University Health Centre, Montréal, Canada; Cleveland Clinic, UNITED STATES

## Abstract

**Introduction and objective:**

Diagnostic testing for *Clostridioides difficile infection* (CDI) by nucleic acid amplification test (NAAT) cannot distinguish between colonization and infection. A positive NAAT may therefore represent a false positive for infection, since diarrhea due to various aetiologies may occur in hospitalized patients. Our objective was to help answer the question: “*does this medical inpatient with diarrhea have CDI*?”

**Design:**

We conducted a retrospective cohort study (n = 248) on the Clinical Teaching Units of the Royal Victoria Hospital (Montréal, Canada). Patients were included if they had a NAAT between January 2014 and September 2015 and their admission diagnosis was not CDI. CDI cases and non-CDI cases were compared, and independent predictors of CDI were determined by logistic regression.

**Results:**

Several factors were independently associated with CDI, including: hemodialysis (OR: 13.5, 95% CI: 2.85–63.8), atrial fibrillation (OR: 3.70, 95% CI: 1.52–9.01), whether the patient received empiric treatment (OR: 3.01, 95% CI: 1.04–8.68), systemic antibiotic therapy prior to testing (OR: 4.23, 95% CI: 1.71–10.5), previous positive NAAT (OR: 3.70, 95% CI: 1.41–9.72), and a leukocyte count of 11x10^9^/L or higher (OR: 3.43, 95% CI: 1.42–8.26). The area under the curve was 0.80.

**Conclusion:**

For patients presenting with hospital-onset diarrhea, various parameters can help differentiate between CDI and other causes. A clinical prediction calculator derived from our cohort (http://individual.utoronto.ca/leet/cdiff.html) might assist clinicians in estimating the risk of CDI for inpatients; those with low pre-test probability may not require immediate testing, treatment, nor prolonged isolation.

## Introduction

Diarrhea is very common among hospitalized patients. There are many causes, including medications, chronic gastrointestinal diseases, as well as acute viral and bacterial infections. Among the latter group, *Clostridioides difficile* (the preferred name for the former *Clostridium difficile* [1] is a leading cause of hospital-onset diarrhea. Since a sudden spike in *C*. *difficile* infection (CDI) rates at the turn of the 21^st^ century in both Canadian and American hospitals, it has increased drastically in prevalence across North America [2–6], recently surpassing methicillin-resistant *Staphyloccocus aureus* (MRSA) as the most common source of nosocomial infection in many American jurisdictions [7].

Classically, CDI occurs when exposure to antibiotics alters the gut flora, leading to overgrowth of *C*. *difficile* and severe diarrhea due to *C*. *difficile* toxin. If untreated, infection progresses to pseudomembranous colitis and toxic megacolon, ultimately resulting in shock. In 2011, 500,000 Americans were diagnosed with and 29 000 patients died of CDI, costing an estimated US $4.8 billion in acute treatment alone [8].

The diagnosis of CDI remains a matter of scientific debate as there is not yet a universally accepted test or testing algorithm. For example, though stool cultures can sensitively detect the presence of *C*. *difficile*, they require specific laboratory conditions and expertise, as well as prolonged incubation periods, which have resulted in them falling out of favour as first-line diagnostic tests. A modified toxigenic culture, which not only isolates *C*. *difficile* but also confirms the presence of toxin (thereby reducing the possibility of false positive results), is considered the gold standard for diagnosis. However, toxin detection (and the expertise required for these tests) is not yet widely available, so many centres need other rapid tests to rely on.

Since 2009, highly sensitive *C*. *difficile* toxin nucleic acid amplification tests (NAATs) have therefore emerged as a test of choice in many institutions [9]. These tests primarily detect and amplify the *tcdB* gene, which is responsible for the expression of toxin B, and are thought to be very highly sensitive[10]. However, these assays may also detect the presence of unexpressed *tcdB* genes in patients who do not have disease[11] and may only be asymptomatic carriers of *C*. *difficile*. They must therefore be interpreted in the clinical context, mindful of risk factors like recent antibiotic use, to determine if a patient with a positive assay truly has clinical CDI. Ideally, for diagnostic purposes, they should be sent only in patients with high pre-test probability of CDI.

In particular, for hospitalized patients with multiple comorbidities and concomitant medications, it can be difficult to immediately differentiate CDI from another cause of diarrhea when loose stools develop on the ward. Given the associated costs of the overuse of empiric therapy and challenges due to false-positive testing, we believed that clinicians could benefit from an estimate of the pre-test probability of new onset diarrhea for hospitalized patients being caused by CDI prior to requesting a toxin NAAT.

The objective of our study was therefore to identify clinical and laboratory parameters that were ultimately associated with a confirmed diagnosis of CDI, in order to answer: “*does this medical inpatient with healthcare-facility onset diarrhea have C*. *difficile infection*?”

## Methods and materials

Patient information was obtained from chart review of patients admitted to the medical clinical teaching units (CTU) at the Royal Victoria Hospital in Montréal, Canada. Data was extracted from January 2014 to September 2015. Patients were included in the study if their admission diagnosis was not *C*. *difficile* and if they were tested for *C*. *difficile* by NAAT during their admission. An episode of CDI was defined as a patient with new diarrhea any time after admission and a positive *C*. *difficile* NAAT who received at least ten days of treatment with either oral vancomycin or metronidazole. Our cases are described as healthcare-facility onset diarrhea with acquisition of *C*. *difficile* either in community or at the healthcare institution. At our hospital, in the absence of toxic megacolon or shock, a *C*. *difficile* NAAT is sent when there are three or more unformed stools within 24 hours and all cases were confirmed to meet the standard IDSA-SHEA definition [12] by trained infection control personnel.

Clinical and laboratory data were extracted from patient records. Clinical data notably included medical comorbidities and previous history of positive *C*. *difficile* toxin NAAT (dating back to June 2010, which is when the test entered use at our institution). We also recorded laxative prescription within the last 48 hours, number of bowel movements within 24 hours, as well as charting of abdominal tenderness and the quality of stool within 24 hours. Our hospital protocol states that NAAT tests are, in general, not sent for fewer than three bowel movements in 24 hours. Therefore, patients who did not have a specific documented number of bowel movements were assumed to have less than five but at minimum three bowel movements. Additionally, when abdominal tenderness was not explicitly mentioned in the chart, it was assumed to be absent.

Although laxatives were often held after diarrhea developed, this variable was included to determine if laxative prescription at time of diarrhea onset influenced pre-test probability for CDI. Fever within 24 hours of diarrhea was included and assessed by charted vital signs, which at our institution are measured every eight hours on the inpatient unit. Antibiotic exposure within the previous 30 days was also recorded and assessed by prescription history within our hospital network, or mention of recent antibiotic exposure in the patient’s chart. Empiric therapy was defined as the initiation of *C*. *difficile* therapy prior to the receipt of NAAT results. At least 10 days of *C*. *difficile* directed therapy was considered to be a full course of CDI therapy. The highest white blood cell (WBC) count on the day of sample procurement as well as in the preceding 72 hours was recorded and leukocytosis was defined as a WBC above 11 × 10^9^/L as this is the cut-off in our laboratory. Creatinine levels on the day of testing were also recorded; acute kidney injury was defined as an increase of 26 μmol/mL from a patient’s baseline, excluding patients with end-stage renal disease on dialysis who were considered separately [13]. For both WBC and creatinine, if same-date data were not available, data from within 48 hours before or after the specimen procurement was used.

Ethics approval was granted by the McGill University Health Centre Research Ethics Board who waived the requirement for informed consent given the retrospective nature of our study. After data was extracted, all patient data were de-identified and analyzed in an anonymous fashion.

Univariate comparisons were made using chi-square and multivariate comparisons were made using logistic regression, with a P-value of .05 representing significance. To avoid over-representing patients with multiple admissions and multiple NAAT tests sent over the study period, only the first test result per patient was used in the derivation of the regression model. The initial logistic regression model was selected using backwards selection with a goal of maximizing the c-statistic. We initially started will all covariates in the model and worked backwards by removing those with a p-value above 0.05 until we arrived at the final model. We then attempted to build the model using forwards selection involving all covariates which converged on the same result. We then forced potential confounders such as age (which were not independently associated) into this model to determine if the c-statistic improved. Since it did not, and those covariates were not associated with the outcome of interest, we did not include them in our final selected model. This final model was converted into an interactive Microsoft Excel calculator using the co-efficient for each term in the equation for the post-test probability predicted by regression.

## Results

Of the 2537 admissions to the CTU within our specified time frame, 319 NAAT tests for *C*. *difficile* were performed. These samples were obtained from 248 unique patients who were admitted in the absence of an admission diagnosis of CDI. 215 of these unique tests were negative (87%), whereas 33 (13%) were positive and were classified as having CDI (see Table 1). 2 (0.8%) patients tested positive but did not receive treatment, were considered asymptomatic carriers by the clinical treating teams, and are therefore not included in Table 1.

**Table 1:** Demographics of patients included.

	Cases of CDI N = 33 (%)	Controls N = 215 (%)	P-value
**Characteristic**			
Median age (years) (IQR)	76 (63–82)	70 (57–81)	0.07
Female (%)	13 (39%)	113 (53%)	0.14
**Comorbid Diagnoses:**			
Hypertension	24 (72%)	116 (54%)	0.05
Diabetes Mellitus	10 (30%)	66 (31%)	0.91
Coronary Artery Disease	10 (30%)	56 (26%)	0.63
Atrial Fibrillation	13 (39%)	36 (17%)	<0.01
Congestive Heart Failure	9 (27%)	37 (17%)	0.17
Solid Organ Transplant	3 (9%)	38 (18%)	0.20
Solid or Hematologic Cancer	8 (24%)	47 (38%)	0.12
Cirrhosis	3 (9%)	20 (9%)	1.0
End-Stage Renal Disease on Dialysis	6 (18%)	4 (2%)	<0.01
HIV Infection	1 (3%)	9 (4%)	0.78
**Clinical Features**			
Antibiotic Exposure in Past 30 Days	16 (48%)	47 (22%)	<0.01
Five or more bowel movements	7 (21%)	44 (20%)	0.89
Fever	0 (0%)	0 (0%)	1.0
Abdominal Tenderness	9 (27%)	37 (17%)	0.27
Treated Empirically for CDI	8 (24%)	22 (10%)	0.02
Active Laxative Prescription	8 (24%)	73 (34%)	0.26
Proton Pump Inhibitor Use	19 (57%)	126 (59%)	0.83
Mycophenolate Drugs	2 (6%)	34 (16%)	0.13
Calcineurin Inhibitors	1 (3%)	31 (14%)	0.08
**Laboratory Parameters**			
Previous Positive *C*. *Diff* NAAT	11 (33%)	27 (13%)	0.02
Leukocytosis > = 11x10^9^ cells/mL	20 (60%)	84 (39%)	0.02
Median maximal WBC count (x10^9^ cells/mL) (IQR)	12.9 (10.1–19.2)	9.6 (6.8–13.9)	0.01
Acute Kidney Injury^1^	11 (41%)	69 (33%)	0.41

^1^: patients with end-stage renal disease on hemodialysis were excluded from this measurement.

In our multivariable model, a number of factors were independently associated with CDI in medical inpatients including: receipt of hemodialysis (OR: 13.5, 95% CI: 2.85–63.8), atrial fibrillation (OR: 3.70, 95% CI: 1.52–9.01), whether the patient received empiric treatment (OR: 3.01, 95% CI: 1.04–8.68), receipt of systemic antibiotics in the 30 days prior to the test (OR: 4.23, 95% CI: 1.71–10.5), previous positive NAAT (OR: 3.70, 95% CI: 1.41–9.72), and a leukocyte count of 11x10^9^/L or higher (OR: 3.43, 95% CI: 1.42–8.26) (see Table 2). The presence of fever, abdominal tenderness, acute kidney injury, number of bowel movements, description of the stool and laxative use within 48 hours were not found to be independent predictors of CDI. The area under the receiver-operator curve (c-statistic) for the model was 0.80.

**Table 2:** Clinical and laboratory factors independently associated with CDI.

Factor	Odds ratio	95% Confidence		P-value
Dialysis	13.5	2.85	63.8	0.001
Warrants Empiric Therapy	3.01	1.04	8.68	0.042
Atrial Fibrillation	3.70	1.52	9.01	0.004
Antibiotic exposure ≤ 30 days	4.23	1.71	10.45	0.002
Previous positive *C*. *diff* NAAT	3.70	1.41	9.72	0.008
WBC Count ≥ 11	3.43	1.43	8.26	0.006

We used the results of our study to derive a preliminary clinical prediction calculator (accessible at: http://individual.utoronto.ca/leet/cdiff.html), which converts the coefficients of our regression model into a means by which one could estimate the pre-test CDI probability for individual medical patients. Most non-CDI cases of diarrhea in our cohort were ultimately considered to be related to medication side effects although there were likely other infectious illnesses with diarrhea as a component (influenza and viral gastroenteritis). There were no cases of *de novo* inflammatory bowel disease diagnosed.

## Discussion

Our data suggest that a number of clinical or laboratory findings are associated with an increased risk of CDI among hospitalized patients with diarrhea. Unsurprisingly, recent antibiotic exposure (OR: 4.23, 95% CI: 1.71–10.5), a previous positive NAAT (OR: 3.70, 95% CI: 1.41–9.72) and leukocytosis (OR: 3.43, 95% CI: 1.42–8.26) were associated with increased risk of CDI in our model. These findings corroborate the literature as being important and independent markers of disease.

In addition, our results support the concept that clinical judgment as shown by the belief that the patient requires empiric therapy while diagnostic testing results are pending is a reasonable predictor that the patient is more likely to have CDI than another cause of diarrhea (OR: 3.01, 95% CI: 1.04–8.68). Although this concept is subjective, it reflects the many complexities of clinical judgment, which are difficult to precisely quantify but which have been shown in other clinical prediction rules to add discriminative power [14].

Our data also suggests that dialyzed patients with diarrhea were significantly more likely (OR: 13.5, 95% CI: 2.85–63.8) than patients not on dialysis to have CDI. In our analysis, AKI and creatinine levels were not correlated with increased probability of CDI, suggesting that renal injury was not the inherent predisposing risk factor. Rather, this finding is more likely explained by the fact that dialyzed patients, whose frequent weekly visits over a long time period cumulate to significant hospital exposure, are more likely to be exposed to and acquire *C*. *difficile*. In addition, although it is possible that these patients’ uniquely thorough medical records may cause misclassification bias, their charts are consolidated upon admission to the inpatient unit, which mitigates this risk. As these patients are also relatively immune suppressed, they may also be more likely to experience disease [15].

Atrial fibrillation (OR: 3.70, 95% CI: 1.52–9.01) was also found to increase the likelihood of a patient with diarrhea having CDI. This may be a chance association due to an unmeasured covariate within our cohort or represent an increased risk of CDI due to other metabolic and/or pharmacologic differences for these patients.

Our work has several limitations. First, our study population was restricted to the inpatient clinical teaching units of one institution and our sample size was relatively small so our findings should be interpreted with caution. We did not have enough of a sample to provide a derivation and validation subset and our model requires validation elsewhere. However, at the time of our study, these units had the highest rate of inpatient CDI at our institution and as it is a general medical unit the patients represent a heterogeneous and diverse population. Secondly, we have relied on charted comorbidities, vital signs (including temperature), physical findings, and stool characteristics which may have introduced information bias in cases where this information was incorrectly documented. Moreover, as we only had access to data from our own hospital network, we lacked comprehensive data on a full history of healthcare exposures, previous positive *C*. *difficile* NAAT assays as well as out-of-centre antibiotic use. Nonetheless, the data available in our chart review was the same information that treating teams used when deciding to test for *C*. *difficile* or to treat a positive result. Furthermore, at our institution, admission comorbidities are reviewed at minimum three times (initially by the emergency room physician, the emergency room internal medicine consultant, as well as on admission to the internal medicine unit). They are therefore highly comprehensive at our centre.

Thirdly, although NAAT tests were sent shortly after the onset of diarrhea due to standard operating procedures in our institution, the exact timing relative to symptom onset could not be ascertained due to the retrospective nature of our study It is also worth noting that patients with previous positive NAAT may be more likely to be tested, which may affect our results. Indeed, if CDI colonization status is known (because of prior NAAT results or previous confirmed infection) there could certainly be a bias towards ordering testing in such patients which may bias the results in these cases. A prospective study with structured data collection, assessing information on other risk factors such as the magnitude of health care contact or *C*. *difficile* exposure/colonization pressure, would lead to a superior tool.

Finally, we only evaluated patients whose stool was tested and not all patients with diarrhea in the hospital. Therefore, we may have under-sampled the group of patients that the treating team felt had the lowest probability of CDI. It is possible that not all cases of CDI were identified in our cohort, such that false negatives may have been considered CDI-negative controls. However, the NAAT employed has sensitivity close to 100% [16] and no negative patient was fully treated for CDI.

Our study therefore presents a number of clinical and laboratory findings, outside of the usually well-recognized risk factors, that influence the likelihood of a hospitalized patient with healthcare facility-onset diarrhea having CDI. Importantly, unlike previous work, which compares patients with CDI to those without irrespective of clinical presentation [17, 18], we have attempted to determine who has CDI amongst the more relevant subset of patients with diarrhea. Our results encourage diagnostic stewardship by helping to determine the pre-test probability of CDI in patients with healthcare facility-onset diarrhea, to help clinicians evaluate the utility of a toxin NAAT. To translate our data into an actionable clinical tool, we integrated our results into a model that allows clinicians to enter patient information and determine a likelihood of a patient having CDI. It remains to be seen whether such a rule would be valid in a derivation cohort and if the use of these pre-test probabilities might fit into testing and empiric treatment algorithms.

## Conclusion

Our cohort is the first to specifically examine the clinical prediction of CDI in the setting of new onset diarrhea in medical inpatients. For this specific population, certain clinical and laboratory parameters may be useful to differentiate between those with CDI and other aetiologies. If validated in a future cohort, this clinical prediction rule may adjust the pre-test probability of CDI for this patient population and inform diagnostic and management approaches.
